# Knowledge, Attitude, and Practices of Pregnant Women Towards COVID-19: An On-site Cross-sectional Survey

**DOI:** 10.7759/cureus.27259

**Published:** 2022-07-25

**Authors:** Chanchal Singh, Gazala Shahnaz, Ram Bajpai, Jayasree Sundar

**Affiliations:** 1 Fetal Medicine, Madhukar Rainbow Children's Hospital and BirthRight, Delhi, IND; 2 Fetal Medicine, Madhukar Rainbow Children's Hospital and BirthRight, New Delhi, IND; 3 School of Primary, Community and Social Care, Keele University, Staffordshire, GBR; 4 Obstetrics and Gynaecology, Madhukar Rainbow Children's Hospital and BirthRight, New Delhi, IND

**Keywords:** sars-cov-2, pregnancy, pandemic, maternal mental health, kap study, covid-19

## Abstract

Objective: To assess the knowledge, attitude, and practices (KAP) of pregnant women towards coronavirus disease 2019 (COVID-19).

Methods: This on-site cross-sectional survey was conducted in the antenatal and fetal medicine clinics in a tertiary care hospital in North India. Pregnant women attending the maternal-fetal unit filled out a 31-item questionnaire assessing their KAP towards COVID-19. Correlation between KAP was assessed by using Spearman’s rank correlation.

Results: Some 302 questionnaires were analyzed: more than 90% of women had correct general knowledge of COVID, but only 12.3% scored 50% or more for pregnancy-related knowledge. Some 67% of women reported more than usual anxiety, and 7.7% reported severe anxiety levels. General knowledge improved with age, education, and occupation but pregnancy-related knowledge and anxiety score were unaffected by these variables.

Conclusions: Pregnant women's knowledge of COVID-19 infection, in general, is excellent and they have the correct attitude towards preventive strategies. However, knowledge and attitude towards its effect on pregnancy are limited.

## Introduction

Coronavirus disease 2019 (COVID-19) caused by severe acute respiratory syndrome coronavirus-2 (SARS-Cov-2) was declared a pandemic by the World Health Organization (WHO) in March 2020 [1]. As on 14th March 2021, there have been more than 11.8 million confirmed cases and more than 26 lakh deaths worldwide (https://covid19.who.int). The disease has had an unprecedented impact on routine healthcare facilities including maternity care. Pregnancy is one of the most vulnerable times in a woman's life; dealing with the economic and health aspects of a pandemic in addition to the usual stresses of pregnancy is bound to affect pregnant women even when they may not be COVID positive. Also, any disease of this scale and widespread impact are likely to give rise to misinformation and misconceptions, which in turn lead to uncertainty and fear. Although current evidence states that pregnant women are not more likely to contract the disease than others, and when infected, most will have a good outcome [2-4], this information may not be known to women. Currently, there are limited evidence on the knowledge, attitude, and practices (KAP) of pregnant women towards the pandemic. Considering that there is no effective cure for COVID-19 at present, we have to rely on modifying behavioral practices to tackle the current pandemic [5]. Understanding the KAP of the target group will make this process more scientific. Thus, this on-site survey was undertaken to assess the KAP of pregnant women attending a maternal-fetal unit during the current pandemic.

## Materials and methods

This on-site cross-sectional survey was conducted at a tertiary care hospital in North India. It included all pregnant women attending the antenatal and fetal medicine units from 15th April to 30th June 2020. A questionnaire (Annexure 1) with 31 items was prepared using information available on the World Health Organization (WHO), Royal College of Obstetricians and Gynaecology (RCOG), and Society of Maternal-Fetal Medicine (SMFM) websites [6]. The questionnaire was prepared in both English and in the local language. It was first filled by 10 hospital healthcare workers and 10 patients to assess the ease of understanding. These 20 questionnaires were not included in the study. After incorporating their suggested changes, hard copies of the final questionnaire were kept in the outpatient antenatal and fetal medicine departments, and all pregnant women attending these clinics were offered to fill the questionnaire. Care was taken that one woman filled only one questionnaire. All women filled out the anonymized questionnaire voluntarily and gave written informed consent for the study. There was no compensation linked to the filling of the questionnaire. The study was approved by the Institutional Review Board (ECR/1360/Inst/DL/2020). 

The Core Outcomes in Women’s and Newborn Health (CROWN) initiative lists ‘maternal attitudes toward routines and practices within a model of care’ and ‘maternal mental health’ as core outcome sets that are the focus of this study [7]. 

Sample size calculation

We assumed that around 50% of pregnant women have appropriate KAP for the COVID-19 pandemic. We further considered 90% power and 5% type I error to detect 10% absolute variability. Using one-sample hypothesis testing formula, we required at least 259 pregnant women to be surveyed for their KAP. We further assumed around 10% of eligible women may refuse to participate in this survey. Therefore, the calculated sample size was further adjusted for a 10% refusal rate, and the minimum operational sample size for this study was 285 pregnant women.

Questionnaire and scoring

The questionnaire consists of two parts -- demographic details and the KAP study. There are 14 questions in the 'knowledge' section, half directed towards general knowledge of COVID-19 and the remaining half towards knowledge of its impact on pregnancy. Six questions aim at assessing pregnant women's attitudes toward COVID-19 and 11 questions about their practice in the current situation. Each question has three options as answers: ‘Yes’, ‘No,’ and ‘Do not know’ with one point given to each response; a higher score in any given section indicates better knowledge. The questions related to KAP were highly consistent (Cronbach’s α = 0.821).

A visual analog scale (VAS) was added at the end of the questionnaire to assess pregnant women’s anxiety ranging from 0 (not at all anxious) to 10 (extremely anxious) and referred to the following question: 'how anxious are you regarding the coronavirus epidemic and the possibility of vertical transmission to your offspring?'

Statistical analysis

Continuous data are presented with a mean (±SD, standard deviation) and categorical variables are presented with numbers and proportions. Correlation between KAP was assessed by using Spearman’s rank correlation. Association between background characteristics (age, education, occupation, and anxiety) and KAP was assessed using univariable and multivariable logistic regression. Model estimates were reported as odds ratios (ORs) and 95% confidence intervals (CIs). All tests were two-sided, and the criterion for statistical significance was set at p<0.05. The data were analyzed using the statistical software IBM SPSS (version 26.0, Armonk, NY, USA).

## Results

A total of 505 women attended the antenatal and fetal medicine unit during the study period. Three hundred sixty women filled up the forms giving a response rate of 71.2%. Fifty-eight questionnaires were excluded because they were filled incompletely. The mean age (± SD) of women in the study cohort was 30.9 ± 4.1 years. All women had completed at least secondary education. Some 81.5% had a graduate degree and above. This was consistent with the urban background of our study population. Almost half of them were working (52.3%) and the rest were homemakers (47.7%). Most women (91.4%) women visited the hospital for a scheduled visit; 9% came in an emergency. Some 67% of women reported more than usual anxiety on the VAS scoring. 

Table 1 lists the distribution of scoring on KAP questions. Almost 80% of women gave correct answers to the first seven questions related to general knowledge on COVID-19. Some 93% of women scored more than 50% on these questions. All were aware of the new infection, its main clinical features, and that the cases were increasing. Some 91.1% of women answered correctly that they should avoid going out of their homes and pay more than usual attention to personal hygiene by washing their hands more frequently and using disinfectants and hand sanitizers. Some 82% of women agreed that if they had any exposure, they needed to isolate themselves and keep a watch for any symptoms related to COVID-19.

**Table 1:** Distribution of KAP questions. KAP, knowledge, attitude, and practice

Questions (n = 302)	N (%)
Knowledge	
General (6 questions)	
0 correct answer (none)	4 (1.3)
1-3 correct answers	15 (5.0)
4-5 correct answers	42 (13.9)
6 correct answers (all)	241 (79.8)
Pregnancy-related (6 questions)	
0 correct answer (none)	117 (38.7)
1-3 correct answers	148 (49.0)
4-5 correct answers	32 (10.6)
6 correct answers (all)	5 (1.7)
Patient awareness about the changes in the maternity unit to tackle COVID	
Yes	233 (73.8)
No/no idea	57 (18.9)
Not filled	22 (7.3)
Attitude	
General	
Positive hope of control	177 (58.6)
Contact exposure	27 (7.9)
Concern of infection	148 (49.0)
Pregnancy-related	
Concern of infection to the baby	186 (61.6)
Concern mode of delivery	149 (49.3)
Happy to change in maternity care	220 (72.8)
Practice	
General (2 questions)	
0 correct answer (none)	13 (4.3)
1 correct answer	14 (4.6)
2 correct answer (all)	275 (91.1)
Pregnancy-related	
Response to need for routine care	139 (46)
Isolation and observation	247 (81.8)
Visit after prior information	236 (78.1)
If COVID positive: call the doctor	252 (83.4)
If COVID positive: come to the hospital directly	97 (32.1)
Hand wash	276 (91.4)
Avoid coughing or sneezing	268 (88.7)
Face shield/cover	265 (87.7)
Expressed milk	222 (73.5)

However, only 12.3% of women scored 50% or more for pregnancy-related knowledge: for example, the majority of women (92%) answered correctly that they should avoid going to crowded places or use of public transport but 54% felt that they should still come to the hospital for routine antenatal care and scans during the lockdown.

Given the hypothetical scenario that they become COVID positive, 83% of women answered that they would call their obstetrician. However, one-third (32%) responded that they would come to the hospital directly which is contrary to current guidance. Some 61.6% of women were concerned about the impact of the disease on their pregnancy and answered that the disease is more dangerous in pregnant women with an increased risk of miscarriage, preterm delivery as well as an increase in the risk of structural abnormalities in the unborn child. Some 78% answered that a child born to an infected mother may also have an adverse effect on its development and intelligence. 

Some 91% of women were aware that they should wash their hands before breastfeeding and avoid coughing or sneezing while handling their newborn. Some 87.7% said they would wear a mask while breastfeeding. Some 73.5% of women were aware that they had the option of giving expressed breast milk through an uninfected attendant. 

Some 73.8% of respondents were aware of the changes in maternity units due to the ongoing pandemic. Some 58.6% of women were hopeful that the pandemic would eventually be controlled but a majority of them were still concerned about getting infected.

Table 2 shows the association between knowledge and demographic characteristics. General knowledge improved with age (p=0.004), education (p = 0.002), and occupation (p<0.001), and this improvement was statistically significant; however, pregnancy-related knowledge and anxiety scores were unaffected by these variables. 

**Table 2:** Association between knowledge and demographic characteristics. *Correct general knowledge is considered if score is equal to 6; #Correct pregnancy-related knowledge is considered if score more than or equal to 1.

Characteristics (N = 302)	Knowledge (general)			Knowledge (pregnancy-related)		
	Correct knowledge^*^		p-value	Any correct answer^#^		p-value
	No (%)	Yes (%)		No (%)	Yes (%)	
Age group (years)						
<30	32 (52.5)	78 (32.4)	0.004	45 (38.5)	65 (35.1)	0.558
≥30	29 (47.5)	163 (67.6)		72 (61.5)	120 (64.9)	
Education						
Up to 12th	17 (27.9)	39 (16.2)	0.003	27 (23.1)	29 (15.7)	0.219
Undergraduate	33 (54.1)	105 (43.6)		53 (45.3)	85 (45.9)	
Masters and above	11 (18.0)	97 (40.2)		37 (31.6)	71 (38.4)	
Occupation						
Not working	44 (72.1)	100 (41.5)	p<0.001	57 (48.7)	87 (47.0)	0.774
Working	17 (27.9)	141 (58.5)		60 (51.3)	98 (53.0)	
Anxiety score						
0-2 mild	23 (37.7)	77 (32.0)	0.693	37 (31.6)	63 (34.1)	0.126
3-6 moderate	34 (55.7)	146 (60.6)		67 (57.3)	113 (61.1)	
7-10 severe	4 (6.6)	18 (7.5)		13 (11.1)	9 (4.9)	
Total	61 (20.2)	241 (79.8)		117 (38.7)	185 (61.3)	

The association between background characteristics and attitudes and baby-care practices is given in Table 3. Older and working women had a better attitude (hope of control) though their educational status did not affect their attitude. A lower anxiety score was also associated with a better attitude. These variables did not directly affect baby-care practices. 

**Table 3:** Association between background characteristics and attitude and baby-care practices. 0 represents complete negative attitude; 6 represents complete positive attitude; baby-care practice yes if all four questions are answered yes.

Characteristics (N = 302)	Attitude score		p value	Baby-care practice		p value
	0-2	3-6		No	Yes	
	n (%)	n (%)		n (%)	n (%)	
Age group (years)						
<30	42 (42.0)	68 (33.7)	0.157	35 (41.7)	75 (34.4)	0.240
≥30	58 (58.0)	134 (66.3)		49 (58.3)	143 (65.6)	
Education						
Up to 12th	21 (21.0)	35 (17.3)	0.370	16 (19.0)	40 (18.3)	0.961
Undergraduate	40 (40.0)	98 (48.5)		39 (46.4)	99 (45.4)	
Masters and above	39 (39.0)	69 (34.2)		29 (34.5)	79 (36.2)	
Occupation						
Not working	60 (60.0)	84 (41.6)	0.003	48 (57.1)	96 (44.0)	0.041
Working	40 (40.0)	118 (58.4)		36 (42.9)	122 (56.0)	
Anxiety score						
0-2 mild	42 (42.0)	58 (28.7)	0.006	29 (34.5)	71 (32.6)	0.838
3-6 moderate	47 (47.0)	133 (65.8)		50 (59.5)	130 (59.6)	
7-10 severe	11 (11.0)	11 (5.4)		5 (6.0)	17 (7.8)	
Total	100	202		84	218	

A positive attitude had a statistically significant correlation with correct practices.

## Discussion

COVID-19 has been an unprecedented healthcare crisis of our times. Governments have imposed restrictions of various degrees in terms of 'lockdowns' to minimize the spread of the virus. This has resulted in a significant negative impact on access to routine healthcare due to extreme measures of isolation, physical distancing, restricted transport, and curtailed outpatient services in hospitals. Pregnant women are a vulnerable group both physically and psychologically. The increased risk of infection during mandatory hospital visits also makes this group particularly vulnerable to acquiring infection. Since there is no effective cure, prevention remains the mainstay for controlling the COVID-19 epidemic. Prevention of any infection needs behavior modification which in turn requires an understanding of the KAP of the affected population. Plenty of information is being released daily regarding the disease as well as its impact on pregnancy from premier organizations [2, 6]; however, whether this information is helping its target population in behavior modification is uncertain. Gathering KAP of pregnant women to COVID-19 will also help health organizations in addressing the factors that are causing anxiety and affecting their mental health adversely. Thus this study was undertaken during the initial period of the pandemic to assess the KAP of pregnant women residing in an urban area of North India towards COVID-19. 

Our study group comprises a well-educated population with good access to the internet and healthcare facilities. Participants were reasonably well aware of the disease in general, its symptoms, mode of transmission, and common interventions to prevent its spread. The knowledge score for COVID-19, in general, was 7.5-fold higher as compared to pregnancy-related information. For example, most women (92%) knew they should avoid going to crowded places or using public transport, but more than half (54%) still felt they should come to the hospital for routine antenatal care and scans even during the lockdown period. Although most women were aware that isolation of an infected person is essential to prevent transmission, nearly one-third (32%) answered that they would come to the hospital directly if they found out they were COVID positive, which is contrary to current guidance [2]. 

Despite reassuring data that most infected pregnant women and their newborns will have a good outcome, 61.6% of women answered that the disease is more dangerous in pregnant women with an increased risk of miscarriage and preterm delivery. They also thought that COVID-19 in pregnancy could lead to an increased risk of structural abnormalities in the fetus. Nearly two-thirds expressed concerns regarding the long-term development and intelligence of a child born to an infected mother. These results highlight that concerns among pregnant women regarding COVID’s impact on their pregnancy and newborn remain unanswered despite the availability of pregnancy-related guidelines. This needs to be addressed urgently as the impact of these misconceptions on pregnant women's mental health is significant. Prior studies have demonstrated that the mere formulation of policies does not ensure that vulnerable groups have access to essential services [8] and having an insight into the KAP is essential in bridging this gap. 

As soon as COVID-19 was declared a pandemic, the maternal-fetal unit at our hospital modified its working as per current recommendations [2, 9-11]. Waiting areas, outpatient clinics, and scan rooms have been rearranged to adhere to physical distancing norms. Video-consults and tele-consults have been adopted for routine care. Ultrasounds in fetal medicine units have been prioritized and restricted. No attendant is being allowed inside scan rooms. Healthcare workers wear masks and personal protective equipment (PPE) as needed. Equipment cleaning is being done as recommended. Some 73.8% of women in our study population were aware of these changes, and almost all (72.8%) women took these changes positively. 

This study shows a significant association of knowledge with a positive attitude and practices in general. Some 81.8% of women knew they needed to isolate themselves in case of 'exposure'. Some 78.1% said they would visit the hospital after prior information. The scores on practices while caring for the baby, like frequent hand washing, avoiding coughing/sneezing, and using a mask/face shield, were consistently above 85%. These high scores on knowledge in general and baby-care practices can be attributed to the awareness generated by social media resources. 

A pandemic of this scale compounded by limited access to healthcare can create undue anxiety and adversely affect mental health. Our study shows that the pandemic had a moderate to severe psychological impact on our study population, with more than 67% of women reporting higher than usual anxiety and 7.7% were severely anxious. This is comparable to other studies though literature is still limited [12-15]. One article reports a higher uptake of cell-free DNA in first-trimester risk assessment compared to combined screening and higher cesarean delivery rates on maternal requests. Both decisions are probably influenced by the potential fear of fetal harm [13]. In our cohort, 61.6% of women were concerned about the risk to their fetus, and 49.3% were worried about its impact on their mode of delivery.

Our study shows that a positive attitude is correlated with correct practice; thus, if the gaps in knowledge related to pregnancy are filled, it will translate into better practices. Therefore, we suggest that maternity units formulate locally designed patient information leaflets (PILs) that include local data to ensure that pregnant women get the correct information. They can also provide infographics and audio formats already available on WHO and RCOG websites to women under their care. Health authorities can develop online or smartphone-based applications or provide information in audio form in native, simple languages to support those with no educational background during the epidemic.

The timing of this study is a major strength -- it was conducted at the peak of the disease curve in our state. This was the first time such drastic measures of complete lockdown were imposed. The questionnaire was filled by women physically coming out of the security of their homes and visiting the hospital, facing mental and logistic challenges. 

Limitations of the study include its single-center design and small sample size. However, the limited number reflects the limited access to healthcare during the pandemic's peak. Another limitation is that we could not assess pregnant women's KAP towards vaccination as the study period predated the introduction of COVID vaccines. 

## Conclusions

In conclusion, pregnant women have reasonably good knowledge of COVID-19 infection in general and a correct attitude towards preventive strategies. However, knowledge about COVID-19 in pregnancy is still limited despite the women being educated and having access to social media. Results from this study will help in addressing the lacunae that prevent the reach of relevant information to pregnant women and the enhance provision of psychological support in these vulnerable times.

## Appendices

**Table 4:** KAP questionnaire in English. KAP, knowledge, attitude, and practice

S.N	Knowledge	Response
K1	Are you aware of COVID infection?	a).Yes b).No c). No idea
K2	Main clinical feature of COVID-19 are fever, fatigue, dry cough, and myalgia	a).Yes b).No c).No idea
K3	Cases of COVID-19 disease are increasing in india	a).Yes b).No c). No idea
K4	No effective cure for COVID-19 is currently available, but symptomatic and supportive treatment can help most patients recover from the infection	a).Yes b).No c). No idea
K5	To prevent the infection by COVID-19 we should avoid going to crowded places or use of public transport	a).Yes b).No c). No idea
K6	To prevent the infection by COVID-19, isolation and treatment of people who are infected are necessary	a).Yes b).No c). No idea
K7	Disease is more dangerous in pregnant women	a).Yes b).No c). No idea
K8	COVID may transmit from infected women to their unborn child	a).Yes b).No c). No idea
K9	Newborns are not at high risk of becoming seriously unwell with the virus	a).Yes b).No c). No idea
K10	COVID infection in pregnancy is associated with increased risk of miscarriage	a).Yes b).No c). No idea
K11	COVID infection in pregnancy is associated with increased risk of premature delivery	a).Yes b).No c). No idea
K12	COVID infection in pregnancy is associated with increased risk of congenital malformation	a).Yes b).No c). No idea
K13	Baby development and intelligence	a).Yes b).No c). No idea
K14	Are you aware of changes in maternity units (changes in routine practice to minimize the spread of coronavirus infection to healthy women and their babies, restricting access to visitors, using appropriate protection equipment, and infection control measures)	a).Yes b).No c). No idea
A	Attitude	RESPONSE
A1	Do you agree that COVID-19 will finally be successfully controlled?	a).Yes b).No c). No idea
A2	In recent days, have you gone to any crowded place or had contact with a person with h/o travel/fever/cough/confirmed case of COVID	a).Yes b).No c). No idea
A3	Do you have concerns regarding being infected?	a).Yes b).No c). No idea
A4	Do you have concerns about your baby being affected if you get infected?	a).Yes b).No c). No idea
A5	Are you concerned regarding the mode of delivery?	a).Yes b).No c). No idea
A6	Are you happy with maternity care providers with new guidelines to minimize the risk of infection (Including restricting access to visitors, using appropriate protection equipment, and infection control measures)	a).Yes b).No c). No idea
P	Practice to Prevent Contacting and Spreading COVID	Response
P1	Avoid going out of my home	a).Yes b).No c). No idea
P2	Frequently wash my hands, use disinfectants and solutions, or pay more attention to my personal hygiene than usual	a).Yes b).No c). No idea
P3	Should you attend antenatal and postnatal appointments if well at the moment and have had no complication	a).Yes b).No c). No idea
P4	Will you go for immediate isolation and observation for symptoms, if you have contact with someone infected with COVID-19	a).Yes b).No c). No idea
P5	Will you contact with healthcare provider directly, if symptomatic for COVID-19	a).Yes b).No c). No idea
P6	Will you contact the maternity unit for advice and to agree on a plan via phone or video consultation, if you are positive for coronavirus?	a).Yes b).No c). No idea
P7	Will you contact your maternity unit for advice and to agree on a plan directly, if you are positive for coronavirus?	a).Yes b).No c). No idea
P8	Will you wash your hands before touching your baby, to prevent infection the newborn if infected	a).Yes b).No c). No idea
P9	Will you try to avoid coughing or sneezing on your baby while feeding at the breast	a).Yes b).No c). No idea
P10	Will you consider wearing a face mask while breastfeeding	a).Yes b).No c). No idea
P11	Will you consider asking someone who is well to feed your expressed breast milk to your baby if you are COVID positive	a).Yes b).No c). No idea

**Table 5:** KAP questionnaire in Hindi. KAP, knowledge, attitude, and practice

क्रम संख्या	ज्ञान	प्रतिक्रिया
K 1	क्या आप कोविड संक्रमण से अवगत हैं?	क) हाँ ख) नहीं ग) कोई जानकारी नहीं
K 2	कोविड 19 की मुख्य नैदानिक ​​विशेषता बुखार, थकान, सूखीखाँसी और माइलगिया हैं	क) हाँ ख) नहीं ग) कोई जानकारी नहीं
K 3	भारत में कोविड -19 बीमारी के मामले बढ़ रहे हैं	क) हाँ ख) नहीं ग) कोई जानकारी नहीं
K4	वर्तमान में कोविड19 के लिए कोई प्रभावी इलाज उपलब्धनहीं है, लेकिन रोगसूचक और सहायक उपचार, मरीजों कोसंक्रमण से उबरने में मदद कर सकते हैं	क) हाँ ख) नहीं ग) कोई जानकारी नहीं
K 5	कोविड 19 द्वारा संक्रमण को रोकने के लिए हमें भीड़-भाड़वाली जगहों पर जाने या सार्वजनिक परिवहन के उपयोग सेबचना चाहिए	क) हाँ ख) नहीं ग) कोई जानकारी नहीं
K 6	कोविड 19 द्वारा संक्रमण को रोकने के लिए, संक्रमित लोगोंके अलगाव और उपचार आवश्यक है	क) हाँ ख) नहीं ग) कोई जानकारी नहीं
K7	गर्भवती महिलाओं में बीमारी अधिक खतरनाक है	क) हाँ ख) नहीं ग) कोई जानकारी नहीं
k 8	कोविड, संक्रमित महिलाओं से अपने अजन्मे बच्चेमें स्थानांतरित कर सकता है	क) हाँ ख) नहीं ग) कोई जानकारी नहीं
K 9	नवजात शिशुओं को वायरस के साथ गंभीर रूप से अस्वस्थहोने का उच्च जोखिम नहीं है	क) हाँ ख) नहीं ग) कोई जानकारी नहीं
K10	कोविड संक्रमण में गर्भपात का खतरा बढ़ जाता है	क) हाँ ख) नहीं ग) कोई जानकारी नहीं
K11	कोविड संक्रमण में, समय से पहले प्रसव का खतरा बढ़ जाताहै	क) हाँ ख) नहीं ग) कोई जानकारी नहीं
K12	गर्भावस्था में कोविड संक्रमण से, जन्मजात विकृति के जोखिमबढ़ जाता है	क) हाँ ख) नहीं ग) कोई जानकारी नहीं
K13	गर्भावस्था में कोविड संक्रमण से, बच्चे का विकास और बुद्धिविकृति के जोखिम बढ़ जाता है	क) हाँ ख) नहीं ग) कोई जानकारी नहीं
K14	क्या आप मातृत्व इकाइयों में बदलावों के बारे में जानते हैं(स्वस्थ महिलाओं और उनके बच्चों को कोरोनावायरससंक्रमण के प्रसार को कम करने के लिए नियमित अभ्यास मेंपरिवर्तन, उचित सुरक्षा उपकरण और संक्रमण नियंत्रणउपायों का उपयोग करके आगंतुकों तक पहुंच को प्रतिबंधितकरता है।)	क) हाँ ख) नहीं ग) कोई जानकारी नहीं
A	रवैया	प्रतिक्रिया
A 1	क्या आप सहमत हैं कि कोविड -19 को आखिरकारसफलतापूर्वक नियंत्रित किया जाएगा?	क) हाँ ख) नहीं ग) कोई जानकारी नहीं
A 2	हाल के दिनों में, आप किसी भीड़-भाड़ वाले जगह पर गया याजो कोई भी व्यक्ति बुखार / खांसी / यात्रा की पुष्टि के मामलेके संक्रमित के साथ संपर्क है	क) हाँ ख) नहीं ग) कोई जानकारी नहीं
A 3	क्या आपको संक्रमित होने के बारे में चिंता है	क) हाँ ख) नहीं ग) कोई जानकारी नहीं
A 4	क्या आपको इस बात की चिंता है कि बच्चे संक्रमित होने परप्रभावित हो रहे हैं	क) हाँ ख) नहीं ग) कोई जानकारी नहीं
A 5	क्या आप डिलीवरी के तरीके के बारे में चिंतित हैं	क) हाँ ख) नहीं ग) कोई जानकारी नहीं
ए 6	क्या आप संक्रमण के जोखिम को कम करने के लिए नएदिशानिर्देशों के साथ मातृत्व देखभाल प्रदाता से खुश हैं ( उचित सुरक्षा उपकरण और संक्रमण नियंत्रण उपायों काउपयोग करके आगंतुकों तक पहुंच को प्रतिबंधित करनाशामिल है।)	क) हाँ ख) नहीं ग) कोई जानकारी नहीं
P	अभ्यास- संक्रमण के प्रसार रोकने के लिए	प्रतिक्रिया
P1	घर से बाहर जाने से बचें	क) हाँ ख) नहीं ग) कोई जानकारी नहीं
P 2	बार-बार मेरे हाथ धोएं, कीटाणुनाशक और समाधान काउपयोग करें या सामान्य से अधिक व्यक्तिगत स्वच्छता परध्यान दें	क) हाँ ख) नहीं ग) कोई जानकारी नहीं
P 3	क्या आपको इस समय सामान्य तरह से प्रसवोत्तर औरप्रसवोत्तर नियुक्तियों में भाग लेना चाहिए अगर कोई जटिलतानहीं है	क) हाँ ख) नहीं ग) कोई जानकारी नहीं
P 4	अगर आप कोविड-19 से संक्रमित किसी व्यक्ति के साथसंपर्क है, क्या आप लक्षणों के लिए तत्काल अलगाव औरअवलोकन के लिए जाएंगे	क) हाँ ख) नहीं ग) कोई जानकारी नहीं
P 5	क्या आप स्वास्थ्य देखभाल प्रदाता के साथ सीधे संपर्क करेंगे, यदि कोविद 19 के लिए रोगसूचक	क) हाँ ख) नहीं ग) कोई जानकारी नहीं
P 6	यदि आप कोरोनोवायरस के लिए सकारात्मक हैं , तो क्याआप सलाह के लिए प्रसूति इकाई से संपर्क करेंगी और फोनया वीडियोकॉन्ट्रेशन की योजना पर सहमति व्यक्तकरेंगी?	क) हाँ ख) नहीं ग) कोई जानकारी नहीं
P 7	अगर आप कोरोनावायरस के लिए सकारात्मकहैं , फिर क्या आप सलाह के लिए और किसी योजनासे सहमत होने के लिए अपनी मातृत्व इकाई से सीधे तौरसंपर्क करेंगे?	क) हाँ ख) नहीं ग) कोई जानकारी नहीं
P 8	नवजात संक्रमण को रोकने के अपने, बच्चे को छूने से पहलेअपने हाथ धो लेंगे?	क) हाँ ख) नहीं ग) कोई जानकारी नहीं
P 9	क्या आप स्तन पर दूध पिलाते समय अपने बच्चे को खांसनेया छींकने से बचने की कोशिश करेंगी	क) हाँ ख) नहीं ग) कोई जानकारी नहीं
P 10	क्या आप स्तनपान करते समय फेस मास्क पहनने पर विचारकरेंगी,	क) हाँ ख) नहीं ग) कोई जानकारी नहीं
P 11	अगर आप कोरोनावायरस के लिए सकारात्मकहैं , फिर क्या आप, आप अपने बच्चे के लिए अपनेव्यक्त मां के दूध को पिलाने के लिए किसी और को कहने परविचार करेंगी,	क) हाँ ख) नहीं ग) कोई जानकारी नहीं
